# Resting heart rate and risk of left and right heart failure in 0.5 million Chinese adults

**DOI:** 10.1136/openhrt-2022-001963

**Published:** 2022-05-31

**Authors:** Valirie Ndip Agbor, Yiping Chen, Robert Clarke, Yu Guo, Pei Pei, Jun Lv, Canqing Yu, Liming Li, Zhengming Chen, Derrick Bennett, the China Kadoorie Biobank Collaborative Group

**Affiliations:** 1 Nuffield Department of Population Health, University of Oxford, Oxford, UK; 2 Medical Research Council, Population Health Research Unit (PHRU), University of Oxford, Oxford, UK; 3 National Center for Cardiovascular Diseases, Chinese Academy of Medical Sciences & Peking Union Medical College Fuwai Hospital, Xicheng District, Beijing, China; 4 Department of Epidemiology and Biostatistics, School of Public Health, Peking University Health Science Center, Beijing, China; 5 Centre for Public Health and Epidemic Preparedness and Response, Peking University, Beijing, China

**Keywords:** heart failure, epidemiology, risk factors

## Abstract

**Objectives:**

To compare the shape and strength of the associations of resting heart rate (RHR) with incident heart failure (HF) and pulmonary heart disease (PHD) in Chinese adults.

**Methods:**

The prospective China Kadoorie Biobank recruited >0.5 million adults from 10 geographically diverse regions (5 urban, 5 rural) of China during 2004–2008. After an 11-year follow-up, 6082 incident cases of HF and 5572 cases of PHD, were recorded among 491 785 participants with no prior history of heart disease or use of beta-blockers at baseline. Cox regression yielded HRs for each disease associated with usual RHR after adjustment for confounding factors.

**Results:**

The mean (SD) baseline RHR was 79 (12) (men 78 (12); women 80 (11)) bpm, and these decreased with increasing age (by about 1 bpm per 10 years). Usual RHR showed J-shaped associations with HF and log-linear associations PHD. For HF, each 10 bpm higher usual RHR was associated with an adjusted HR of 1.25 (95% CI 1.17 to 1.34) for RHR>75 bpm. For PHD, each 10 bpm higher RHR was associated with HR of 1.74 (1.67–1.81) across the full range of usual RHR. For HF at RHR>75 bpm but not PHD, the HRs per 10 bpm higher RHR were approximately halved by further adjustment for diabetes and hypertension.

**Conclusions:**

RHR was strongly positively associated with PHD throughout the range studied, but was only associated with HF at RHR>75 bpm, and the strength of the associations with HF were only one-third of those with PHD.

**WHAT IS ALREADY KNOWN ON THIS TOPIC**

- Resting heart rate (RHR) is a well-established therapeutic target for the treatment of heart failure (HF), but the relevance of heart rate for pulmonary heart disease (PHD) has not been widely studied.
- In the Framingham Heart Study, each 10 bpm higher RHR was associated with 14% higher risk of HF at RHR greater than 80 bpm.

**WHAT THIS STUDY ADDS**

- In an 11-year prospective study of 0.5 million Chinese adults, we examined the shape and strength of the associations of RHR with incident cases of HF and PHD.
- Among those without prior history of heart diseases or use of beta-blockers, usual RHR showed a J-shaped with incident HF and log-linear associations with PHD, respectively.
- Each 10 bpm higher RHR was associated with 25% higher risk of HF at RHR >75 bpm, and with 74% higher risk of PHD throughout the full range of RHR studied.

**HOW THIS STUDY MIGHT AFFECT RESEARCH, PRACTICE AND/OR POLICY**

- Many physicians are reluctant to use beta-blockers in patients with chronic obstructive pulmonary disease (COPD), but this study provides support for more research on the use of beta-blockers for treatment of PHD and prevention of cardiac complications of COPD.

## Introduction

Heart failure (HF), which affects about 25 million people worldwide, is a clinical syndrome arising from structural or functional cardiac abnormalities that result in low cardiac output and increased pressure in the relevant cardiac chambers.^1–5^ Left HF is characterised by the inability of the left ventricle to supply sufficient blood to the systemic circulation and right HF or pulmonary heart disease (PHD) by an inability of the right ventricle to adequately supply the pulmonary circulation. Ischaemic heart disease, hypertensive heart disease and cardiomyopathy account for most cases of left HF and chronic respiratory diseases account for most cases of PHD.^2^

The incidence of HF has increased steadily in recent decades, reflecting improvements in survival following acute cardiac events, longer life expectancy and an increased prevalence of hypertension, diabetes mellitus and obesity.^4 6 7^ HF is also associated with high risks of recurrent hospital admissions, increased healthcare expenditure and substantial in-hospital mortality depending on the healthcare setting.^5 8 9^ PHD has been less widely studied than HF but is believed to account for 6%–7% of all heart disease cases in the USA.^10^

Higher levels of resting heart rate (RHR) have been associated with higher risks of cardiovascular disease (CVD), but the relevance of RHR for both left and right HF is uncertain.^11^ Higher RHR is associated with higher levels of oxidative stress, endothelial dysfunction and atheromatous plaque formation.^12^ Previous studies conducted in mainly Western populations have reported conflicting results, both qualitatively and quantitatively, about the associations of RHR with HF,^13–16^ with some studies reporting J-shaped associations between RHR and HF.^15 16^ It is unclear the extent to which the associations of RHR with HF may be an artefact of incomplete adjustment for confounding by established risk factors, reverse causality or both. Little is known about the associations between RHR and incident HF and PHD in Chinese populations, where the distribution of major risk factors for HF and PHD differs from those in western populations.^5 17^ The aims of the present study were: (i) to compare the shape and strength of the associations of usual RHR with incident HF and PHD, overall and in different population subgroups and (ii) assess the extent to which the observed associations were explained by residual confounding or reverse causality bias.

## Methods

This study was reported in accordance with the Strengthening The Reporting Of Observational studies in Epidemiology (STROBE) guidelines for cohort studies.^18^

### Study design, setting and population

The China Kadoorie Biobank (CKB) is a prospective study of 512 726 Chinese adults who were aged 30–74 years at enrolment in 2004–2008.^19^ The design of the CKB study has been previously described.^19–21^ Briefly, participants were recruited from 10 regions in China. All permanent residents with no major disability in each of the 100–150 administrative units (comprising either urban residential areas or rural villages) were identified through official residential records and invited to participate in the study, with a response rate of about 30%.^20^ Less than 0.5% (n=2470) of individuals who attended the baseline survey were excluded because they withdrew their consent or had missing data. For this study, participants with a self-reported prior history of coronary heart disease (n=16 345) or rheumatic heart disease (n=937) or who reported use of beta-blockers (n=6512) at baseline were excluded. Additional analyses also excluded individuals with a prior history of chronic obstructive pulmonary disease (COPD) and other chronic diseases at baseline.

### Exposures, covariates and outcomes

RHR was measured using calibrated UA-779 digital BP monitor (Omron UA-779; Live Source) to the nearest one beat per minute (bpm) after the participant had been at rest in a seated position for at least 5 min.^19^ RHR was measured twice, and the mean of both measurements was used for the present analyses.

Information on demographic and socioeconomic status (SES), lifestyle, medical history and self-reported health and current use of medication was collected using an interviewer-administered electronic questionnaire at local assessment clinics. All participants had mean levels of weight, height, RHR and blood pressure (BP) recorded. Repeat surveys of random samples of 5% of the CKB population (with about 80% response rates) were conducted at 4 years (2008) and 8 years (2013–2014) after baseline to assess the reproducibility of exposure variables.

Information on vital status and causes of death was collected by linkage, through unique national identification numbers, to death registries at China’s Disease Surveillance Points system, supplemented by annual active follow-up using local residential records, and contacting participants’ family members.^19^ Information on hospital admissions was collected by linkage to the health insurance (HI) claims system for all hospital admissions.^19^ Over 97% of the CKB population reported having HI coverage, and study records are updated every 6 months.^19^ For the small number of uninsured participants, information on hospital admissions was identified through annual active follow-up. All incident fatal and non-fatal disease outcomes were coded, using the International Classification of Diseases tenth revision (ICD-10), by trained staff who were blinded to the baseline information of study participants. In the present report, the analyses were censored at 31 December 2017. The diagnoses of HF and PHD were obtained from HI billing records, death or disease registries that followed contemporary Chinese clinical guidelines for the diagnosis of these diseases. The current clinical guidelines for HF in China include relevant symptoms (dyspnoea on exertion, orthopnoea, fatigue and oliguria) and clinical signs (bilateral pedal oedema, S3 gallop, hepatojugular reflux and pulmonary crepitations) and use of relevant medications (diuretics and angiotensin-converting enzyme II blockers).^22^ The current clinical guidelines for diagnosis of PHD include relevant symptoms, clinical signs and specific findings of enlarged P waves and right ventricular hypertrophy on electrocardiography and echocardiography.^23^ The participants with incident HF and PHD were defined as first non-fatal hospital admission or death due to HF (ICD-10: I50) or PHD (I27).

### Statistical methods

Mean values and SD were used to summarise normally distributed variables, and medians and IQR were used for non-normally distributed variables. Multivariable Cox PH regression models were used to examine the associations of RHR with incident HF and PHD after stratification by region, age-at-risk, sex and season at recruitment. The multivariable regression analyses were sequentially adjusted for SES (marital status, highest level of education and household income in Yuan) and other established CVD risk factors (alcohol consumption, smoking status, physical activity in Metabolic Equivalent of Task hours per day (MET-hour/day), body mass index (BMI, in kg/m^2^), hypertension and diabetes mellitus). Detailed information regarding the categorisation of the covariates included in the multivariable Cox regression analysis is provided in online supplemental table. The likelihood ratio tests (LRT) for heterogeneity were used to assess departures from linearity in continuous variables or ordinal categorical variables. The LRT for heterogeneity was used to assess changes in the χ^2^ statistic for the associations of RHR with incident HF or PHD after sequential addition of potential confounding factors.^24^

10.1136/openhrt-2022-001963.supp1 Supplementary data

All associations of RHR with disease outcomes were corrected for regression dilution bias, by estimating the HRs and their corresponding 95% CI for fifths of baseline RHR and these were plotted against the mean baseline resurvey RHR in the respective baseline-defined groups. The quintile-specific HR and 95% CI were estimated using the floating absolute risk method,^25^ which enabled comparisons of the risks of HF or PHD between any two quintiles of RHR. Moreover, in the setting of a linear association of RHR with HF or PHD, the log HRs per 10 bpm higher baseline RHR (and their associated SEs) were divided by the regression dilution ratio (RDR) to obtain HRs per 10 bpm higher usual RHR.^26^ The RDR was computed using the MacMahon Peto method.^26 27^

Interaction terms were fitted to assess possible effect modification by age (at baseline), sex, smoking, BMI, physical activity, hypertension and diabetes and LRT were used to assess possible effect modification. Sensitivity analyses excluded first events of HF and PHD that occurred during the first 5 years of follow-up or individuals with any prior non-vascular diseases at baseline (chronic obstructive pulmonary disease, emphysema, bronchitis, pulmonary tuberculosis, asthma, any cancer, kidney disease, cirrhosis or hepatitis B or rheumatoid arthritis) or participants with poor self-reported health status at baseline. The impact of additional exclusions of participants with prior stroke or use of BP-lowering medications was also assessed. In addition, we excluded cases of PHD secondary to first HF diagnosis. Similarly, cases of HF after PHD diagnosis were also excluded. Two-tailed p values less than 0.05 were considered statistically significant. Data were analysed using Stata V.16.1 and R V.4.1.

### Patient and public involvement

Local community leaders in China were consulted prior to enrolment of study participants in CKB. The findings of the CKB study are reported in peer-review publications and any relevant public health messages are disseminated using local press, television and internet to study participants.

## Results

**Figure 1:**
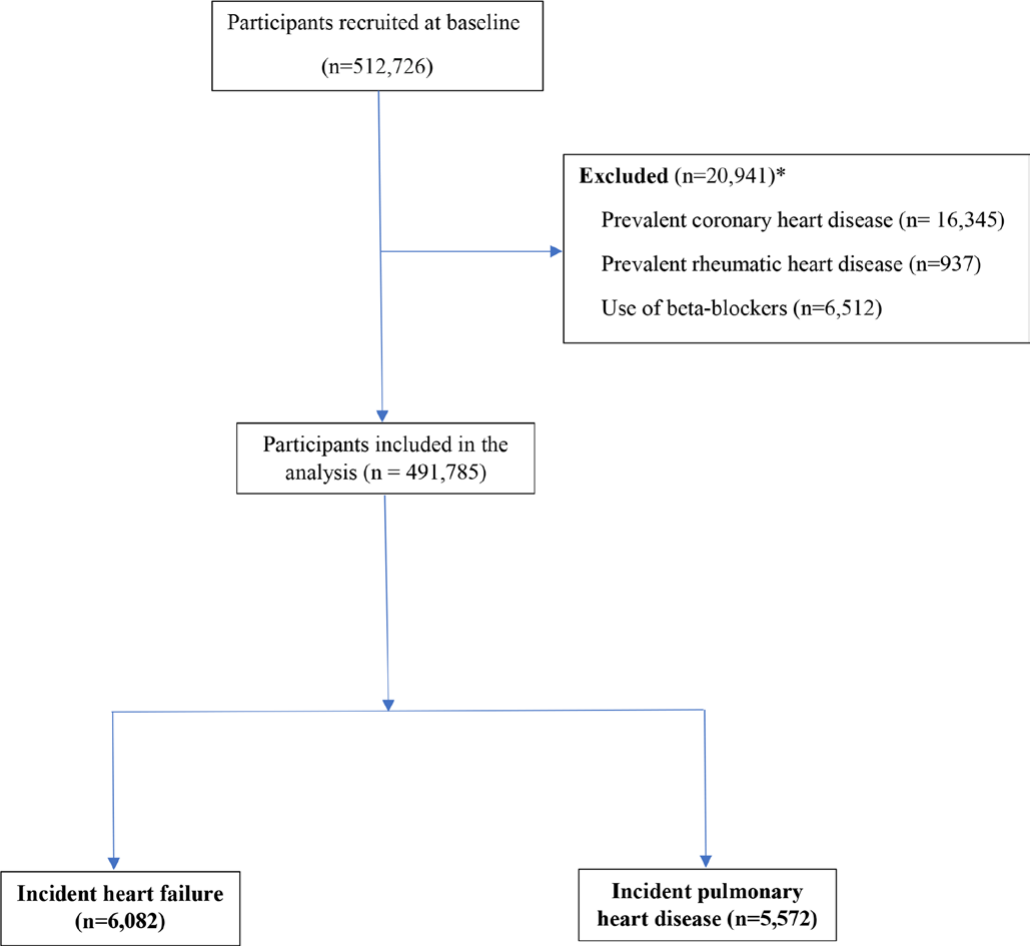
Flow sheet for selection of the study participants. *Categories are not mutually exclusive and, hence, do not add up to 20 941.

Selected baseline characteristics are shown for 491 785 eligible study participants classified by quintiles of RHR in Table 1. Figure 1 shows the flowchart for selection of the study participants. The mean (SD) age was 51.7 (10.6) years, and 60% were women (table 1). Overall, the baseline mean (SD) RHR was 79 (12) bpm (men: 78 (12); women 80 (11)), and RHR declined progressively with increasing age (by 0.76 bpm per 10-year increase). Individuals who were current smokers, less physically active or overweight or had hypertension, obesity or diabetes mellitus had higher mean RHR compared with those without such risk factors (table 1).

**Table 1:** Baseline characteristics of all study participants by fifths of resting heart rate

Characteristic	Fifths of resting heart rate, beats per minute (bpm), (range)					Total
	Q1 (35–69)	Q2 (69.5–75)	Q3 (75.5–81)	Q4 (81.5–88)	Q5 (88.5–198)	
Number of participants, n	100 873	101 540	100 155	93 331	95 886	491 785
Demographic and SES						
Age, mean (SD), year	52.9 (10.5)	51.8 (10.5)	51.4 (10.5)	51.1 (10.6)	51.0 (10.8)	51.7 (10.6)
Women, n (%)	49 680 (49.3)	60 251 (59.3)	61 882 (61.8)	58 312 (62.5)	59 238 (61.8)	289 363 (58.8)
Married, n (%)	91 870 (91.1)	92 476 (91.1)	90 973 (90.8)	84 848 (90.9)	86 539 (90.3)	446 706 (90.8)
Rural area, n (%)	56 384 (55.9)	57 891 (57.0)	54 880 (54.8)	53 531 (57.4)	56 548 (59.0)	279 234 (56.8)
High school education or higher, n (%)	21 113 (20.9)	21 337 (21.0)	21 560 (21.5)	19 269 (20.6)	18 451 (19.2)	101 730 (20.7)
Annual household income >30 000 Yuan, n (%)	44 377 (44.0)	43 499 (42.8)	43 677 (43.6)	38 980 (41.8)	39 112 (40.8)	209 645 (42.6)
Lifestyle factors						
Regular alcohol drinkers, n (%)	17 555 (17.4)	14 972 (14.7)	13 902 (13.9)	13 356 (14.3)	14 350 (15.0)	74 135 (15.1)
Current smoker, n (%)	36 565 (36.2)	30 641 (30.2)	28 852 (28.8)	26 788 (28.7)	27 807 (29.0)	150 653 (30.6)
Physical activity, median (IQR), MET-hour/day	18.4 (10.7–31.3)	18.3 (11.0–30.7)	17.8 (10.7–30.3)	17.7 (10.7–30.2)	17.4 (10.3–29.9)	18.0 (10.7–30.5)
Physical measurements						
Resting heart rate, mean (SD), bpm	64.1 (4.1)	72.4 (1.7)	78.2 (1.8)	84.5 (2.0)	96.7 (7.6)	78.7 (11.7)
SBP, mean (SD), mm Hg	129.2 (21.2)	128.7 (20.5)	129.5 (20.5)	130.9 (20.8)	135.2 (21.6)	130.6 (21.1)
DBP, mean (SD), mm Hg	75.4 (10.6)	76.3 (10.7)	77.3 (10.9)	78.4 (11.0)	81.1 (11.5)	77.7 (11.1)
BMI, mean (SD), kg/m^2^	23.4 (3.2)	23.5 (3.2)	23.6 (3.3)	23.7 (3.4)	23.7 (3.6)	23.6 (3.4)
Self-reported health and disease						
Hypertension, n (%)	31 921 (31.6)	30 140 (29.7)	31 638 (31.6)	31 710 (34.0)	40 691 (42.4)	166 100 (33.8)
Diabetes mellitus, n (%)	3290 (3.3)	4031 (4.0)	5123 (5.1)	5826 (6.2)	8555 (8.9)	26 825 (5.5)
Kidney disease, n (%)	1424 (1.4)	1418 (1.4)	1380 (1.4)	1296 (1.4)	1259 (1.3)	6777 (1.4)
Stroke or TIA, n (%)	1570 (1.6)	1391 (1.4)	1454 (1.5)	1332 (1.4)	1439 (1.5)	7186 (1.5)
Any cancer, n (%)	464 (0.5)	431 (0.4)	456 (0.5)	464 (0.5)	583 (0.6)	2398 (0.5)
COPD, n (%)	7039 (7.0)	6757 (6.7)	6619 (6.6)	6657 (7.1)	7882 (8.2)	34 954 (7.1)
Emphysema or bronchitis, n (%)	2237 (2.2)	2254 (2.2)	2239 (2.2)	2334 (2.5)	3170 (3.3)	12 234 (2.5)
Asthma, n (%)	452 (0.4)	444 (0.4)	461 (0.5)	514 (0.6)	681 (0.7)	2552 (0.5)
Tuberculosis, n (%)	1447 (1.4)	1439 (1.4)	1318 (1.3)	1350 (1.4)	1420 (1.5)	6974 (1.4)
Cirrhosis / Hepatitis B, n (%)	1363 (1.4)	1288 (1.3)	1169 (1.2)	1093 (1.2)	995 (1.0)	5908 (1.2)
Rheumatoid arthritis, n (%)	1969 (2.0)	1970 (1.9)	1979 (2.0)	1744 (1.9)	1783 (1.9)	9445 (1.9)
Poor self-rated health, n (%)	8704 (8.6)	9106 (9.0)	9055 (9.0)	9427 (10.1)	11 389 (11.9)	47 681 (9.7)
Medication use						
BP-lowering drug, n (%)	10 348 (10.3)	8946 (8.8)	9328 (9.3)	9133 (9.8)	11 356 (11.8)	49 111 (10.0)
Aspirin, n (%)	641 (7.2)	556 (6.7)	607 (6.7)	604 (6.6)	693 (6.0)	3101 (6.6)
Statin, n (%)	142 (0.1)	144 (0.1)	160 (0.2)	156 (0.2)	171 (0.2)	773 (0.2)

ACE, angiotensin-converting enzyme; BMI, body mass index; COPD, chronic obstructive pulmonary disease; DBP, diastolic blood pressure; MET, metabolic equivalent task; n, frequency; Q1–5, first to the fifth quintile; SBP, systolic blood pressure; SES, socioeconomic status; TIA, transient ischaemic attack.

### Association of usual resting heart rate with incident heart failure and pulmonary heart disease

**Figure 2:**
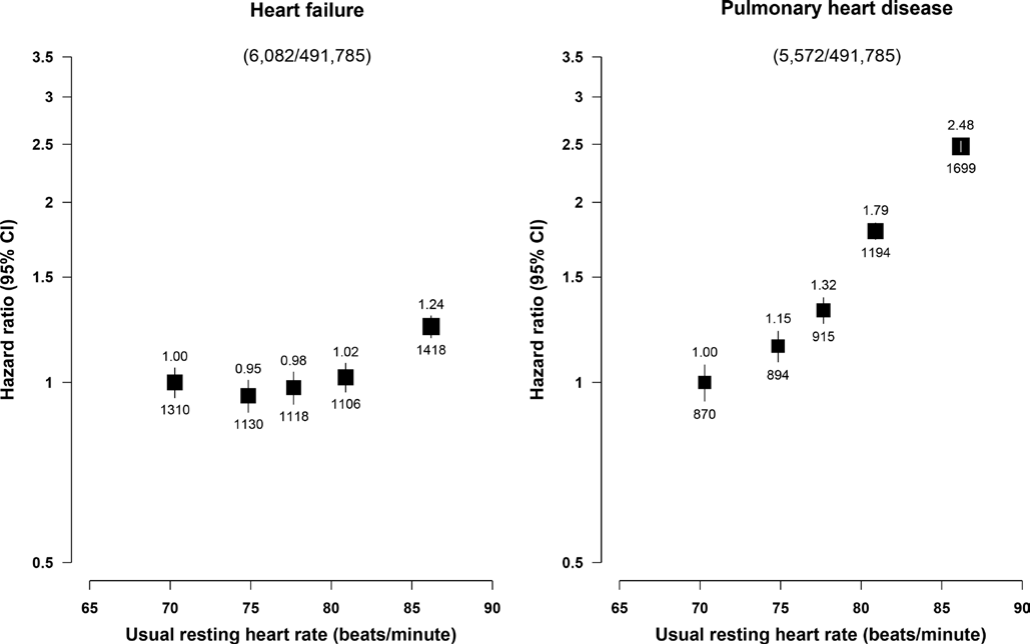
Associations of fifths of RHR with heart failure and pulmonary heart disease. Models were adjusted for measured confounders (season, sex, socioeconomic status (education, household income and marital status), lifestyle (physical activity, smoking and alcohol consumption), body mass index, hypertension and diabetes mellitus) and stratified by age and region. The HR of HF or PHD for each fifth of usual RHR were compared with those in the lowest fifth. The black squares and the vertical bars are adjusted HR and 95% CI. The numbers above and below the vertical bars represent the adjusted HR and number of events in each quintile, respectively. The black squares were weighted by the number of events in each fifth. HF, heart failure; PHD, pulmonary heart disease; RHR, resting heart rate.

During a median follow-up of 11.1 years (IQR: 10.2–12.1) years, 6082 participants had HF and 5572 had PHD, respectively. A total of only 263 (4.7%) of the 5572 PHD occurred following a diagnosis of HF and only 325 (5.2%) of HF cases occurred after a diagnosis of PHD. There was a J-shaped association of RHR with HF, with a weak inverse association at RHR <75 bpm, but an approximately log-linear positive association at RHR ≥75 bpm (figure 2). In contrast, RHR showed strong positive and apparently log-linear association with risk of incident PHD throughout the range studied (figure 2).

**Figure 3:**
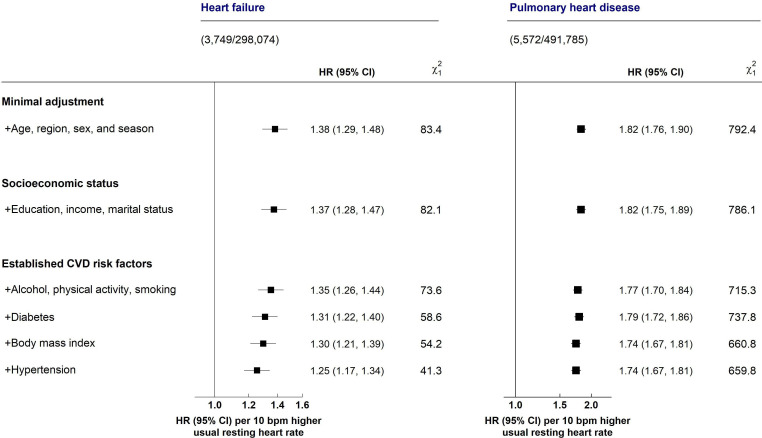
HRs (95% CI) of heart failure and pulmonary heart disease associated with 10 bpm higher RHR before and after adjusting for confounding factors. The squares represent the adjusted HR of HF or PHD per 10 beats per unit (bpm) higher usual RHR. Symbols and conventions as in figure 2. In the left panel, the analyses were restricted at a usual RHR ≥75 bpm, where the shape of the association of usual RHR with HF was approximately linear. CVD, cardiovascular disease; HF, heart failure; RHR, resting heart rate.

Figure 3 shows the effect of sequential adjustment for potential confounding factors on the strength of the associations of RHR with HF (top panel) or PHD (bottom panel). After correction for regression dilution bias, each 10 bpm higher usual RHR was associated with a 25% (1.25; 95% CI 1.17 to 1.34) higher risk of HF for RHR for 75 bpm or greater (figure 3). The strength of the association of RHR with HF was approximately halved after adjusting for confounding due to hypertension and diabetes (as demonstrated by a 50% reduction in the χ^2^_1_ from 83.4 to 41.3). For PHD, each 10 bpm higher usual RHR was associated with a 74% (1.74; 1.67–1.81) higher risk of PHD (figure 3). In contrast with HF, the strength of the association of usual RHR with PHD was largely unaltered by sequential additional adjustment for confounding factors (16.7% reduction in χ^2^_1_: figure 3).

**Figure 4:**
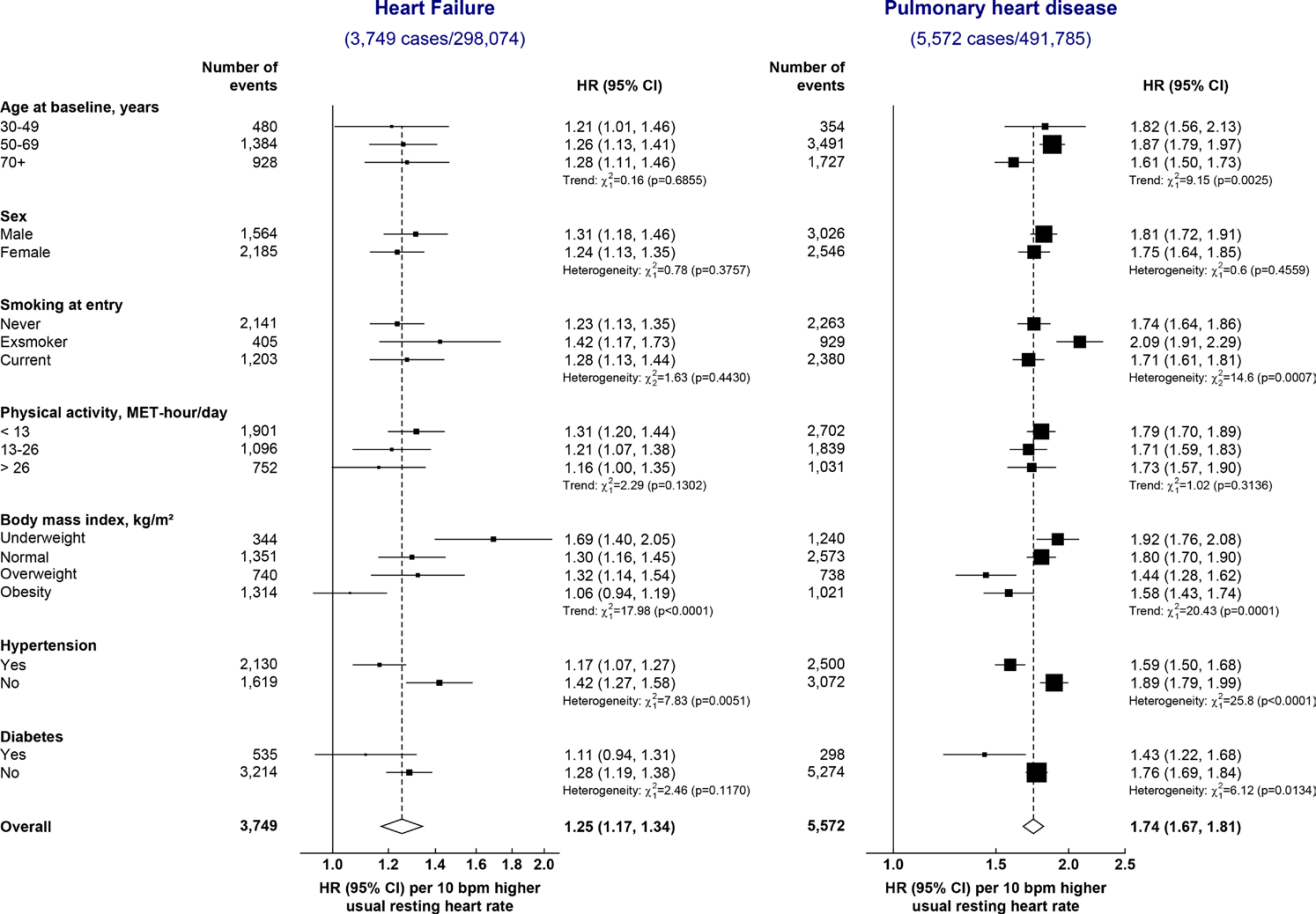
HRs (95% CI) of heart failure and pulmonary heart disease associated with 10 bpm higher resting heart in different population subgroups. Models were adjusted for measured confounders. The unshaded diamond and dashed vertical line represent the overall adjusted HR for the main model, while the solid vertical line represents the line of no effect.

The risks of HF and PHD for each 10 bpm higher usual RHR varied substantially in different subgroups (figure 4). The strengths of the associations of usual RHR with HF were weaker among participants with hypertension and higher BMI, whereas for PHD, they were stronger among ex-smokers, at younger age and in those without diabetes (figure 4).

Online supplemental figure 1 shows the shapes of the associations of fifths of usual RHR with HF and PHD after excluding participants with prior diseases. Table 2 shows the HR (95% CI) of HF and PHD associated with 10 bpm higher RHR after excluding participants with prior diseases. For both HF and PHD, excluding individuals with non-vascular diseases significantly attenuated the associations, while the converse was true after excluding those with prior stroke or BP-lowering medications. Exclusion of non-vascular diseases at baseline reduced the strength of the association of RHR with HF and PHD by about 50% but did not materially change the shape of the associations (online supplemental figure 1 and table 2). In addition, excluding the HF events after a diagnosis of PHD attenuated the association of RHR with HF (table 2 and online supplemental figure 1). However, excluding events occurring during the first 5 years of follow-up did not materially alter the associations.

**Table 2:** HRs (95% CI) of heart failure and pulmonary heart disease associated with 10 bpm higher resting heart rate after excluding participants with prior diseases

Outcome	Events (n)	HR* (95% CI)
Heart failure		
Main model	3749	1.25 (1.17 to 1.34)
Excluding prior non-vascular disease†	2634	1.14 (1.05 to 1.24)
Excluding poor self-rated health	3727	1.18 (1.09 to 1.28)
Excluding the first 5 years of follow-up	2745	1.23 (1.14 to 1.33)
Excluding prior stroke and those on aspirin, and BP-lowering medication	2929	1.31 (1.21 to 1.41)
Excluding HF cases secondary to first PHD diagnosis	3511	1.20 (1.12 to 1.29)
Pulmonary heart disease		
Main model	5572	1.74 (1.67 to 1.81)
Excluding prior non-vascular disease†	2397	1.39 (1.30 to 1.48)
Excluding poor self-rated health	3824	1.59 (1.51 to 1.67)
Excluding the first 5 years of follow-up	3699	1.59 (1.51 to 1.68)
Excluding prior stroke and those on aspirin, and BP-lowering medication	5307	1.79 (1.72 to 1.87)
Excluding PHD cases secondary to first HF diagnosis	5309	1.75 (1.68 to 1.83)

n=frequency. All analyses were adjusted for age-at-risk, sex, season, region, socioeconomic factors (education, household income and marital status); CVD risk factors (alcohol consumption, physical activity, smoking, systolic blood pressure, body mass index and self-reported diagnosis of diabetes mellitus and hypertension diagnosed by a medical doctor). All analyses were stratified by age-at-risk and region.

The risk of heart failure per 10 bpm higher usual resting heart rate was only estimated for usual RHR≥75 bpm where the shape of the association of usual resting heart rate with heart failure was approximately linear.

*All HRs are per 10 beats per minute higher usual heart rate.

†Prior non-vascular disease (chronic obstructive pulmonary disease, emphysema, bronchitis, pulmonary tuberculosis, asthma, any cancer, rheumatoid arthritis, chronic hepatitis or cirrhosis and chronic kidney disease).

BP, blood pressure; PHD, pulmonary heart disease.

## Discussion

This large prospective study in China demonstrated striking differences in associations of RHR with left and right HF. For left HF, there were J-shaped associations of RHR with HF, with the modestly strong positive associations evident only at RHR >75 bpm. Moreover, further adjustment for established CVD risk factors and exclusion of prior non-vascular diseases greatly attenuated the associations towards the null. In contrast, RHR showed strong log-linear positive associations with risks of PHD throughout the range of RHR studied, which were largely unaffected by further adjustment for CVD risk factors. Among participants with RHR >75 bpm, each 10 bpm higher usual RHR was associated with a 25% higher risk of HF but was associated with a 74% higher risk of PHD throughout the range studied.

Previous studies in Western populations have reported that higher RHR was associated with a higher risk of HF in healthy adults and in patients with heart disease or hypertension in both middle-aged and older adults.^16 28–32^ The results of the present study are consistent with those of previous studies in Western populations that reported that higher levels of RHR were only a modest risk factor for HF but a stronger risk factor for PHD.^29 30^ The strength of associations of RHR with HF differed by levels of established CVD risk factors and was stronger among individuals with versus without obesity and hypertension.

The present study highlights the importance of adjusting for confounding by levels of established CVD risk factors when assessing the associations of RHR and HF. Consistent with previous reports, approximately half of the association of RHR with HF was accounted for by hypertension, adiposity and diabetes. Moreover, the present study highlights the importance of reverse causality as the strength of the associations were attenuated after excluding participants with prior non-vascular diseases and individuals with poor self-reported health at baseline. However, the strength of the associations between RHR and PHD was not attenuated after adjustment for confounding factors to the same extent as with HF after adjustment for confounding factors, suggesting that confounding factors were less important for PHD than for HF.

HF is a disease of insidious onset and has a progressive course, with an early asymptomatic stage followed by a progressive reduction in cardiac function and a corresponding increase in heart rate due to activation of the sympathetic nervous system.^32^ The analyses sought to minimise the effects of reverse causality by excluding participants with a prior history of coronary heart disease or rheumatic heart disease or the use of beta-blockers at baseline. Additional exclusions of participants with prior non-vascular disease and individuals with self-reported poor health substantially attenuated the strength of the associations of RHR with HF by over 50%.

The association of RHR with PHD could possibly reflect some dysfunction of the sympathetic nervous system and related peripheral chemoreceptors in response to the vasodilatory effects of hypoxaemia. Increased sympathetic nerve activity could result in elevated RHR, increased stroke volume and systemic vasoconstriction.^2 33^ Prolonged chronic vasoconstriction of the pulmonary arteries and increased cardiac output could result in right ventricular remodelling and failure.

While the study, involving a large number of cases, demonstrated precision in the strength of the associations with both right and left HF, it also had some important limitations. Participants were not advised to avoid smoking or consumption of tea and coffee before examination. Even though the associations between RHR with HF and PHD were corrected for regression dilution bias, it was not possible to correct for within-person variability in covariates.^34^ Moreover, due to the large size of the study, it was not feasible to collect data on heart rhythm at baseline in the CKB. Furthermore, it was not possible to classify HF and PHD by major subtypes as medical records providing details of the diagnostic criteria for major subtypes were not available in CKB. Consequently, we cannot exclude the possibility of some misclassification, as cases of HF and PHD in the CKB were not validated at the time of this analysis. Moreover, we were unable to refute the possibility of some misclassification of COPD as PHD, as both diagnoses are difficult to distinguish in clinical practice. In addition, it is possible that there was some misclassification from failure to diagnose mild cases of HF and PHD. Likewise, it was not possible to fully exclude the possibility of residual confounding (eg, systematic inflammatory markers and use of beta-adrenergic stimulators) and unknown confounders on the associations of RHR with HF and PHD. Finally, the findings of this study cannot be generalised to the Chinese population, as the CKB is not representative of the Chinese population.

## Conclusion

Higher levels of RHR (above 75 bpm) were only modestly and positively associated with higher risks of HF, but were much more strongly associated with higher risks of PHD (across the full range of RHR). This study suggests that RHR may be more strongly associated with right rather than with left HF. RHR is a potentially important modifiable risk factor for HF, since measurements of RHR are readily accessible for both patients and healthcare workers. While use of medication to control heart rate has been shown to reduce hospitalisation and death among selected patients with HF, the relevance of medication or other strategies to control heart rate for prevention of incident HF or PHD is uncertain. The findings of the present study imply that reducing high levels of RHR could significantly reduce morbidity and mortality associated with HF and PHD at a population level. However, further research is needed to assess the causal relevance of the observed associations between RHR with HF and PHD and clinical relevance of using RHR to predict individuals at high risk of HF or PHD based on their RHR.

## Data Availability

Data are available on reasonable request.

## References

[1] Ponikowski P , Voors AA , Anker SD , et al . 2016 ESC Guidelines for the diagnosis and treatment of acute and chronic heart failure: The Task Force for the diagnosis and treatment of acute and chronic heart failure of the European Society of Cardiology (ESC)Developed with the special contribution of the Heart Failure Association (HFA) of the ESC. Eur Heart J 2016;37:2129–200. 10.1093/eurheartj/ehw128 27206819

[2] Forfia PR , Vaidya A , Wiegers SE . Pulmonary heart disease: the heart-lung interaction and its impact on patient phenotypes. Pulm Circ 2013;3:5–19. 10.4103/2045-8932.109910 23662171 PMC3641739

[3] Ambrosy AP , Fonarow GC , Butler J , et al . The global health and economic burden of hospitalizations for heart failure: lessons learned from hospitalized heart failure registries. J Am Coll Cardiol 2014;63:1123–33. 10.1016/j.jacc.2013.11.053 24491689

[4] Conrad N , Judge A , Tran J , et al . Temporal trends and patterns in heart failure incidence: a population-based study of 4 million individuals. Lancet 2018;391:572–80. 10.1016/S0140-6736(17)32520-5 29174292 PMC5814791

[5] Agbor VN , Ntusi NAB , Noubiap JJ . An overview of heart failure in low- and middle-income countries. Cardiovasc Diagn Ther 2020;10:244-251. 10.21037/cdt.2019.08.03 32420107 PMC7225422

[6] Benjamin EJ , Blaha MJ , Chiuve SE , et al . Heart Disease and Stroke Statistics-2017 update: a report from the American Heart Association. Circulation 2017;135:e146–603. 10.1161/CIR.0000000000000485 28122885 PMC5408160

[7] Celermajer DS , Chow CK , Marijon E , et al . Cardiovascular disease in the developing world: prevalences, patterns, and the potential of early disease detection. J Am Coll Cardiol 2012;60:1207–16. 10.1016/j.jacc.2012.03.074 22858388

[8] Agbor VN , Essouma M , Ntusi NAB , et al . Heart failure in sub-Saharan Africa: a contemporaneous systematic review and meta-analysis. Int J Cardiol 2018;257:207–15. 10.1016/j.ijcard.2017.12.048 29506693

[9] Lesyuk W , Kriza C , Kolominsky-Rabas P . Cost-of-illness studies in heart failure: a systematic review 2004-2016. BMC Cardiovasc Disord 2018;18:74. 10.1186/s12872-018-0815-3 29716540 PMC5930493

[10] Garrison DM , Pendela VS , Memon J . Cor Pulmonale. In: StatPearls. Treasure Island (FL): StatPearls Publishing, 2021. 28613490

[11] Fox KM , Ferrari R . Heart rate: a forgotten link in coronary artery disease? Nat Rev Cardiol 2011;8:369–79. 10.1038/nrcardio.2011.58 21519356

[12] Custodis F , Schirmer SH , Baumhäkel M , et al . Vascular pathophysiology in response to increased heart rate. J Am Coll Cardiol 2010;56:1973–83. 10.1016/j.jacc.2010.09.014 21126638

[13] Ho JE , Larson MG , Ghorbani A , et al . Long‐term cardiovascular risks associated with an elevated heart rate: the Framingham heart study. J Am Heart Assoc 2014;3:e000668. 10.1161/JAHA.113.000668 24811610 PMC4309047

[14] Parikh KS , Greiner MA , Suzuki T , et al . Resting heart rate and long-term outcomes among the African American population. JAMA Cardiology 2017;2:172. 10.1001/jamacardio.2016.3234 27681113 PMC5310994

[15] Opdahl A , Ambale Venkatesh B , Fernandes VRS , et al . Resting heart rate as predictor for left ventricular dysfunction and heart failure. J Am Coll Cardiol 2014;63:1182–9. 10.1016/j.jacc.2013.11.027 24412444 PMC4037739

[16] Nanchen D , Stott DJ , Gussekloo J , et al . Resting heart rate and incident heart failure and cardiovascular mortality in older adults: role of inflammation and endothelial dysfunction: the PROSPER study. Eur J Heart Fail 2013;15:581–8. 10.1093/eurjhf/hfs195 23250912

[17] Zhang Y , Zhang J , Butler J , et al . Contemporary epidemiology, management, and outcomes of patients hospitalized for heart failure in China: results from the China heart failure (China-HF) registry. J Card Fail 2017;23:868–75. 10.1016/j.cardfail.2017.09.014 29029965

[18] STROBE statement. Available: https://www.strobe-statement.org/?id=available-checklists [Accessed 03 Dec 2020].

[19] Chen Z , Lee L , Chen J , et al . Cohort profile: the Kadoorie study of chronic disease in China (KSCDC). Int J Epidemiol 2005;34:1243–9. 10.1093/ije/dyi174 16131516

[20] Chen Z , Chen J , Collins R , et al . China Kadoorie Biobank of 0.5 million people: survey methods, baseline characteristics and long-term follow-up. Int J Epidemiol 2011;40:1652–66. 10.1093/ije/dyr120 22158673 PMC3235021

[21] Chen Z , ed. Population Biobank Studies: A Practical Guide. Singapore: Springer Singapore, 2020.

[22] Yu Y , Gupta A , Wu C , et al . Characteristics, Management, and Outcomes of Patients Hospitalized for Heart Failure in China: The China PEACE Retrospective Heart Failure Study. J Am Heart Assoc 2019;8:e012884. 10.1161/JAHA.119.012884 31431117 PMC6755852

[23] You L , Niu H , Huang K , et al . Clinical features and outcomes of acute exacerbation in chronic obstructive pulmonary disease patients with pulmonary heart disease: a multicenter observational study. Int J Chron Obstruct Pulmon Dis 2021;16:2901–10. 10.2147/COPD.S325925 34712043 PMC8547596

[24] Floud S , Balkwill A , Canoy D , et al . Social participation and coronary heart disease risk in a large prospective study of UK women. Eur J Prev Cardiol 2016;23:995–1002. 10.1177/2047487315607056 26416995 PMC4871172

[25] Easton DF , Peto J , Babiker AG . Floating absolute risk: an alternative to relative risk in survival and case-control analysis avoiding an arbitrary reference group. Stat Med 1991;10:1025–35. 10.1002/sim.4780100703 1652152

[26] MacMahon S , Peto R , Cutler J , et al . Blood pressure, stroke, and coronary heart disease. Part 1, prolonged differences in blood pressure: prospective observational studies corrected for the regression dilution bias. Lancet 1990;335:765–74. 10.1016/0140-6736(90)90878-9 1969518

[27] Frost C , Thompson SG . Correcting for regression dilution bias: comparison of methods for a single predictor variable. J R Stat Soc Ser A Stat Soc 2000;163:173–89. 10.1111/1467-985X.00164

[28] Pfister R , Michels G , Sharp SJ , et al . Resting heart rate and incident heart failure in apparently healthy men and women in the EPIC-Norfolk study. Eur J Heart Fail 2012;14:1163–70. 10.1093/eurjhf/hfs104 22736739

[29] Khan H , Kunutsor S , Kalogeropoulos AP , et al . Resting heart rate and risk of incident heart failure: three prospective cohort studies and a systematic meta-analysis. J Am Heart Assoc 2015;4:e001364. 10.1161/JAHA.114.001364 25589535 PMC4330063

[30] Butler J , Kalogeropoulos A , Georgiopoulou V , et al . Incident heart failure prediction in the elderly: the health ABC heart failure score. Circ Heart Fail 2008;1:125–33. 10.1161/CIRCHEARTFAILURE.108.768457 19777072 PMC2748334

[31] Kannel WB , D'Agostino RB , Silbershatz H , et al . Profile for estimating risk of heart failure. Arch Intern Med 1999;159:1197–204. 10.1001/archinte.159.11.1197 10371227

[32] Reil J-C , Custodis F , Swedberg K , et al . Heart rate reduction in cardiovascular disease and therapy. Clin Res Cardiol 2011;100:11–19. 10.1007/s00392-010-0207-x 20809390

[33] Schultz HD , Li YL , Ding Y . Arterial chemoreceptors and sympathetic nerve activity: implications for hypertension and heart failure. Hypertension 2007;50:6–13. 10.1161/HYPERTENSIONAHA.106.076083 17502495

[34] Greenland S . The effect of misclassification in the presence of covariates. Am J Epidemiol 1980;112:564–9. 10.1093/oxfordjournals.aje.a113025 7424903

