# Supplementary material for: Resting heart rate and risk of left and right heart failure in 0.5 million Chinese adults
Source: Open Heart. 2022 May 31;9(1):e001963. doi: 10.1136/openhrt-2022-001963 (PMC9161067; doi:10.1136/openhrt-2022-001963)

Supplementary material for:

Resting heart rate and risk of left and right heart failure in 0.5 million Chinese adults

Table of contents

Members of the CKB Collaborative Group Pages 2-3

Supplementary Table: Variables included in the Cox regression analysis Page 5

Supplementary Figure: Associations of fifths of resting heart rate with heart failure and pulmonary heart disease after excluding participants with prior diseases Page 5

Agbor VN, *et al. Open Heart* 2022; 9:e001963. doi: 10.1136/openhrt-2022-001963

Members of the China Kadoorie Biobank Collaborative Group

International Steering Committee: Junshi Chen[6], Zhengming Chen (PI)[1,5], Robert Clarke[1], Rory Collins[1], Yu Guo[4], Liming Li (PI)[5], Jun Lv[5], Richard Peto[1], and Robin Walters[1].

International Co-ordinating Centre, Oxford: Daniel Avery[1], Derrick Bennett[1], Ruth Boxall[1], Fiona Bragg[1], Sushila Burgess[1], Kahung Chan[1], Yumei Chang[1], Yiping Chen[1,3], Zhengming Chen[1], Robert Clarke[1], Huaidong Du[1], Zammy Fairhurst-Hunter[1], Wei Gan[1], Simon Gilbert[1], Alex Hacker[1], Parisa Hariri[1], Michael Holmes[1], Andri Iona[1], Becky Im[1], Maria Kakkoura[1], Christiana Kartsonaki[1,3], Rene Kerosi[1], Kuang Lin[1], Iona Millwood[1], Qunhua Nie[1], Alfred Pozaricki[1], Paul Ryder[1], Sam Sansome[1], Dan Schmidt[1], Rajani Sohoni[1], Rebecca Stevens[1], Iain Turnbull[1], Robin Walters[1], Lin Wang[1], Neil Wright[1], Ling Yang[1], Xiaoming Yang[1] and Pang Yao[1].

National Co-ordinating Centre, Beijing: Yu Guo[2], Xiao Han[2], Can Hou[2], Chao Liu[2], Jun Lv[3,4], Pei Pei[2], Canqing Yu[3],[4] and Liming Li[3,4].

Regional Co-ordinating Centres: **Qingdao** CDC: Zengchang Pang[7], Ruqin Gao[7], Shanpeng Li[7], Shaojie Wang[7], Yongmei Liu[7], Ranran Du[7], Liang Cheng[7], Xiaocao Tian[7], Hua Zhang[7], Yaoming Zhai[7], Feng Ning[7], Xiaohui Sun[7], Feifei Li[7]. Licang CDC: Silu Lv[8], Junzheng Wang[8], Wei Hou[8]. **Heilongjiang** Provincial CDC: Mingyuan Zou[9], Shichun Yan[9], Xue Zhou[9]. Nangang CDC: Bo Yu[10], Yanjie Li[10], Qinai Xu[10], Quan Kang[10], Ziyan Guo[10]. **Hainan** Provincial CDC: Ximin Hu[11], Jinyan Chen[11], Xiaohuan Wang[11]. Meilan CDC: Min Weng[12], Zhendong Guo[12], Shukuan Wu[12], Yilei Li[12], Huimei Li[12]. **Jiangsu** Provincial CDC: Ming Wu[13], Yonglin Zhou[13], Jinyi Zhou[13], Ran Tao[13], Jie Yang[13], Jian Su[13]. Suzhou CDC: Fang Liu[14], Jun Zhang[14], Yihe Hu[14], Yan Lu[14], Liangcai Ma[14], Aiyu Tang[14], Yujie Hua[14], Jianrong Jin[14], Jingchao Liu[14]. **Guangxi** Provincial CDC: Zhenzhu Tang[15], Naying Chen[15], Duo Liu[15]. Liuzhou CDC: Mingqiang Li[16], Jinhuai Meng[16], Rong Pan[16], Qilian Jiang[16], Jian Lan[16], Yun Liu[16], Liuping Wei[16], Liyuan Zhou[16], Ningyu Chen[16], Ping Wang[16], Fanwen Meng[16], Yulu Qin[16], Sisi Wang[16]. **Sichuan** Provincial CDC: Xianping Wu[17], Ningmei Zhang[17], Xiaofang Chen[17], Xunfu Zhong[17], Jiaqiu Liu[17], Qiang Sun[17]. Pengzhou CDC: Guojin Luo[18], Jianguo Li[18], Xiaofang Chen[18], Xunfu Zhong[18], Jiaqiu Liu[18], Qiang Sun[18]. **Gansu** Provincial CDC: Pengfei Ge[19], Xiaolan Ren[19], Caixia Dong[19]. Maiji CDC: Hui Zhang[20], Enke Mao[20], Zhongxiao Li[20], Tao Wang[20], Xi Zhang[20]. **Henan** Provincial CDC: Ding Zhang[21], Gang Zhou[21], Shixian Feng[21], Liang Chang[21], Lei Fan[21]. Huixian CDC: Yulian Gao[22], Tianyou He[22], Huarong Sun[22], Pan He[22], Chen Hu[22], Xukui Zhang[22]. **Zhejiang** Provincial CDC: Min Yu[23], Ruying Hu[23], Hao Wang[23], Weiwei Gong[23], Meng Wang[23]. Tongxiang CDC: Chunmei Wang[24], Xiaoyi Zhang[24], Kaixu Xie[24], Lingli Chen[24], Dongxia Pan[24], Qijun Gu[24]. **Hunan** Provincial CDC: Yuelong Huang[25], Biyun Chen[25], Li Yin[25], Huilin Liu[25], Zhongxi Fu[25], Qiaohua Xu[25]. Liuyang CDC: Xin Xu[26], Hao Zhang[26], Huajun Long[26], and Libo Zhang[26].

Affiliations:

[1] Clinical Trial Service Unit and Epidemiological Studies, Nuffield Department of Population Health, University of Oxford, UK

[2] Department of Engineering Science, University of Oxford, UK

[3] Medical Research Council, Population Health Research Unit, University of Oxford, UK

[4] Chinese Academy of Medical Sciences, Beijing, China

[5] Department of Epidemiology and Biostatistics, School of Public Health, Peking University

Health Sciences Center, Beijing, China, Peking University Center for Public Health and Epidemic Preparedness & Response, Beijing, China

[6] China National Center For Food Safety Risk Assessment, Beijing, China

[7] Qingdao CDC, Qingdao, Shangdong, China

[8] Licang CDC, Licang District, Qingdao, Shandong, China

[9] Heilongjiang CDC, Harbin, Heilongjiang, China

[10] Nangang CDC, Nangang District, Harbin, Heilongjiang, China

[11] Hainan CDC, Haikou, Hainan, China

[12] Meilan CDC, Meilang District, Haikou, Hainan, China

[13] Jiangsu CDC, Nanjing, Jiangsu, China

[14] Suzhou CDC, Suzhou, Jiangsu, China

[15] Guangxi CDC, Nanning, Guangxi, China

[16] Liuzhou CDC, Liuzhou, Guangxi, China

[17] Sichuan CDC, Chengdu, Sichuan, China

[18] Pengzhou CDC, Pengzhou, Sichuan, China

[19] Gansu CDC, Lanzhou, Gansu, China

[20] Maiji CDC, Maiji, Tianshui, Gansu, China

[21] Henan CDC, Zhengzhou, Henan, China

[22] Huixian CDC, Huixian, Henan, China

[23] Zhejiang CDC, Hanzhou Zhejiang, China

[24] Tongxiang CDC, Tongxiang, Zhejiang, China

[25] Hunan CDC, Changsha, Hunan, China

[26] Liuyang CDC, Liuya

Table: Variables included in the Cox regression analysis

Covariates	Groups
**Demographic and socioeconomic factors**	
**Age-at-risk**	Five-year bands
**Sex**	Male and female
**Region**	Represents the ten regions of the China Kadoorie Biobank (CKB)
**Area**	This was categorised into rural and urban areas. The ten CKB regions include five urban and five rural areas.
**Season at recruitment**	Four seasons: Winter, Spring, Autumn, and Summer
**Marital status**	Four groups: Never married, married, separated or divorced, and widowed
**Highest level of education**	No formal education, primary education, middle school, and high school or higher
**Annual household income in Yuan**	Four groups: <10,000, 10,000 – 29,999, 30,000 – 34,999, or ≥ 35,000
**Established cardiovascular disease risk factors**	
**Physical activity**	Three groups: Tertiles of physical activity measured in Metabolic Equivalent of Task hours per day
**Smoking**	Three groups: Never smoker, ex-smoker smoker, and current smoker
**Alcohol consumption**	Five groups: never regular drinker, ex-regular drinkers, reduced intake, occasional drinkers, and regular drinkers
**Body mass index (in kg/m^2)**	Four groups: Underweight, normal weight, overweight and obese
**Hypertension**	Two groups: Presence or absence of hypertension. Hypertension was defined as either a history of physician-diagnosed hypertension or the use of blood pressure (BP)-lowering medications or a systolic BP ≥140 mmHg or a diastolic BP ≥90 mmHg measured at baseline survey.
**Diabetes**	Two groups: Diabetes and no diabetes

Reference: *Chen et al., China Kadoorie Biobank of 0.5 million people: survey methods, baseline characteristics and long-term follow-up, International Journal of Epidemiology, Volume 40, Issue 6, December 2011, Pages 1652–1666, https://doi.org/10.1093/ije/dyr120*

Supplementary Figure 1: Associations of fifths of resting heart rate with heart failure and pulmonary heart disease after excluding participants with prior diseases

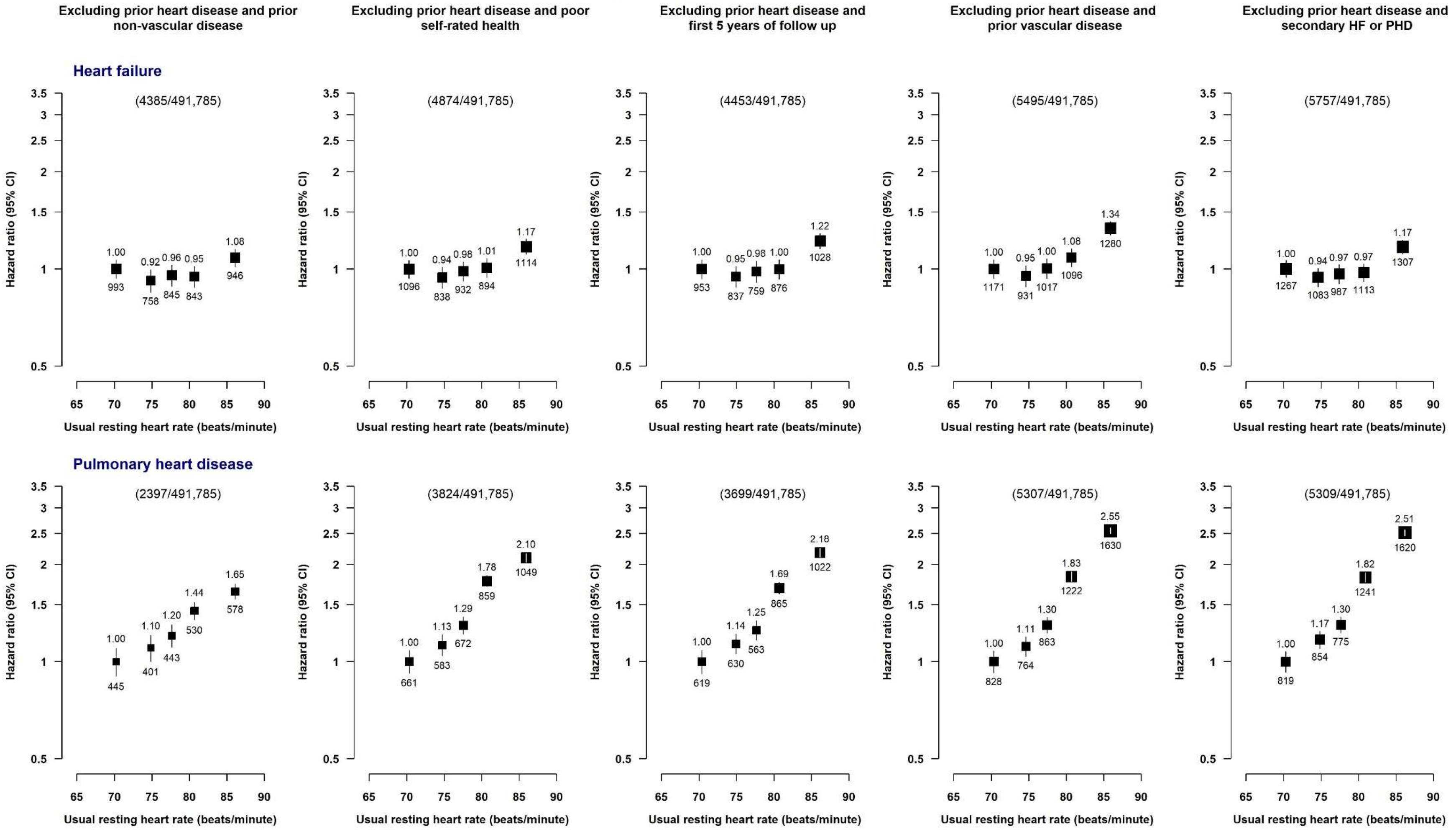

Supplement: Supplementary data [file openhrt-2022-001963supp001.pdf]
